# Fothergillian Prize Essays for 1837 and 1838

**Published:** 1836-07

**Authors:** 


### FOTHERGILLIAN PRIZE ESSAYS FOR 1837 AND 1838.

The Council of the Medical Society of London have selected the following subjects for essays for the Fothergillian medal:—For March, 1837, "On the Use and Abuse of Mercury;" for March, 1838, "The Structure, Economy, and Diseases of the Ear." The Fothergillian medal is valued at twenty guineas. It may be competed for by any one on the following conditions: 1. The essay must be sent to the Registrar on or before the 1st day of December. 2. With it shall be delivered a sealed packet, with some motto or device on the outside, and within the author's name and designation; and the same motto or device shall be put on the dissertation, that the Society may know how to address the successful candidate. 3. No paper in the handwriting of the author can be received.

